# Abdominal Aortic Calcification as a Predictor of Incomplete Adjuvant Chemotherapy in Stage III Colorectal Cancer: A Retrospective Cohort Study

**DOI:** 10.7759/cureus.71288

**Published:** 2024-10-12

**Authors:** Kouki Imaoka, Manabu Shimomura, Hiroshi Okuda, Takuya Yano, Shintaro Akabane, Masahiro Ohira, Yuki Imaoka, Tetsuya Mochizuki, Minoru Hattori, Hideki Ohdan

**Affiliations:** 1 Department of Gastroenterological and Transplant Surgery, Graduate School of Biomedical and Health Science, Hiroshima University, Hiroshima, JPN; 2 Advanced Medical Skills Training Center, Institute of Biomedical and Health Sciences, Hiroshima University, Hiroshima, JPN

**Keywords:** adjuvant chemotherapy, calcification, clinical frailty, colorectal cancer, incompletion

## Abstract

Objectives

Completion of postoperative adjuvant chemotherapy (AC) contributes to the improved prognosis of patients with pathological stage (pStage) III colorectal cancer (CRC). Therefore, identifying patients with AC intolerance is important. Although abdominal aortic calcification (AAC) indicates frailty, its clinical impact on AC completion remains unclear. This study aimed to clarify the association between AAC and AC incompletion.

Methods

Patients who underwent AC for pStage III CRC between 2010 and 2021 (n = 161) were enrolled in this study. AAC volume was evaluated by preoperative CT tomography, and the patients were divided into two groups based on an AAC cutoff of 992 mm^3^, determined using the receiver operating characteristic curves for AC completion. We investigated the perioperative clinicopathological factors and compared the frequency and severity of AC adverse events between the groups.

Results

The high AAC group had a significantly higher proportion of patients with older age, male sex, hypertension, and AC incompletion than the low AAC group. The regimens were not significantly different. No significant difference in the frequency or severity of adverse events was observed in either group. In the multivariate analysis, high AAC and older age were significantly associated with AC incompletion. Furthermore, k-means cluster analysis based on both age and AAC volume also demonstrated an increased risk of AC incompletion in patients with stage III CRC as both age and AAC volume increased. High AAC was associated with diminished improvement in nutritional status or inflammatory markers after the administration of AC.

Conclusions

High AAC is a potential risk marker for predicting AC incompletion in patients with stage III CRC before introducing AC.

This article was previously posted to the Research Square preprint server on May 16, 2024.

## Introduction

Colorectal cancer (CRC) is the third most commonly diagnosed cancer and the second leading cause of cancer-related deaths globally [1]. Adjuvant chemotherapy (AC) plays a crucial role in mitigating postoperative recurrence and improving patient prognosis [2,3]. Particularly, AC completion has been linked to reduced rates of postoperative recurrence, establishing it as the standard treatment protocol for stage III CRC [3]. However, the adverse effects (AEs) of chemotherapy can be more pronounced in patients with comorbidities or deteriorating general health [4]. Consequently, factors such as advanced age and pre-existing health conditions are frequently cited as reasons for non-initiation of AC [5]. Considering these observations, it is critical to identify the risk factors that impede the completion of AC before its initiation. This process can facilitate the adjustment or moderation of chemotherapy intensity or necessitate more stringent monitoring of patients undergoing AC.

Abdominal aortic calcification (AAC) is known to be correlated with increased mortality related to cardiovascular diseases [6]. AAC is also associated with malnutrition, chronic inflammation, and atherosclerosis syndrome [7,8]. Recent studies have indicated that the severity of vascular calcification exhibits a linear positive relationship with frailty in older adults [9], with older women at risk for rapid weight loss over a five-year period [10]. While AAC can simply be a marker of advanced age and frailty, there are several putative mechanisms by which AAC can potentially influence the risk of AC toxicity. Firstly, AAC has been reported to be associated with postoperative renal dysfunction [11]. Therefore, AAC could affect the pharmacokinetics of renally metabolized drugs, such as fluoropyrimidine and oxaliplatin. In addition, advanced age is correlated with a decline in bone marrow reserve and an increased risk of complications related to chemotherapy-induced myelosuppression [12]. Given such associations, we hypothesized that AAC could be an indicative marker of patient-specific vulnerability, thus acting as a reliable predictor of AC incompletion due to AC toxicity in stage III CRC patients. This study explored the efficacy of AAC as an indicator of AC incompletion.

## Materials and methods

Patients

Patients with CRC who underwent radical surgery and were diagnosed with pathological stage (pStage) III CRC in our institute between 2010 and 2021 were included. Clinical data at the time of colorectal surgery, including age, sex, American Society of Anesthesiologists Physical Status Classification System (ASA-PS) score [13], body mass index, hypertension (HT), diabetes mellitus (DM), hyperlipidemia (HL), blood examination data, postoperative complications, and pathological findings, were collected retrospectively from the medical records of the patients. In addition, the availability of AC introduction, details of the regimens, continuation or discontinuation, and severity of AEs were investigated. The decision of AC discontinuation was based on a comprehensive assessment by the physician, considering the degree of AEs, the patient's overall condition, and wishes; the patient did not complete the intended course as scheduled at the time of induction, even after the specified dose was reduced by two levels or to a minimal dose level; the physician judged that the protocol treatment was difficult to continue; and the patient requested discontinuation of protocol treatment. This analysis excluded patients who did not undergo AC, patients who relapsed during the AC period, and patients who were not followed up during the AC period. For the postoperative adjuvant therapy, oxaliplatin combination therapy (capecitabine plus oxaliplatin or fluorouracil plus oxaliplatin) or fluoropyrimidine monotherapy was selected according to the guidelines of the Japanese Society for Cancer of the Colon and Rectum [14], considering the patient's postoperative condition and risk of recurrence. In this cohort study, no patients received capecitabine plus oxaliplatin for a planned three-month period. Clinical data after AC introduction, including blood examination results or the degree of AEs, were collected retrospectively from the medical records of the patients. The patients were followed up using contrast-enhanced computed tomography (CT) and colonoscopy, combined with an evaluation of serum CEA and CA19-9 levels at three-month intervals for five years and at six-month intervals for five years thereafter. This study was conducted in accordance with the guidelines of the Declaration of Helsinki (Fortaleza, Brazil, October 2013) and was authorized in advance by the institutional review board of the Hiroshima University Hospital (approval no. E-744-3).

Evaluation of AAC

Preoperative CT tomography was conducted using a 320-detector row CT scanner (Aquilion ONE ViSION, Toshiba Medical Systems, Otawara City, Japan) with a standardized examination protocol. The AAC score was determined utilizing the AZE Virtual Place Lexus64 Anatomia software (AZE Inc., Tokyo, Japan). Employing the Agatston method [15], the software automatically computed the volume of AAC by identifying calcifications within the abdominal aorta, spanning from the origin of the renal artery to the iliac bifurcation. The AAC cutoff (992 mm^3^) was determined using the receiver operating characteristic curves of AC completion.

Statistical analysis

Categorical variables are expressed as numbers and percentages. Continuous variables are presented as medians with interquartile ranges. Data from the two groups were compared using the chi-squared test or Fisher’s exact test for dichotomous outcomes. Paired Student's t-tests and the nonparametric Mann-Whitney U test were used for continuous outcomes with a skewed distribution. Univariate analyses were performed to assess the association between AC discontinuation and the following variables: age, sex, ASA-PS, AAC, HT, HL, DM, modified Glasgow prognostic score (mGPS) [16], Geriatric Nutritional Risk Index (GNRI) [17], controlling nutritional status (CONUT) [18], neutrophil-to-lymphocyte ratio (NLR) [19], tumor location, postoperative complications, and pStage. All variables were included in a multivariate logistic regression model using a stepwise (forward or backward) procedure. Independent variables were entered into the model at the 0.10 significance level and removed at the 0.20 level; these were used to select the covariates. Based on the results from indicators of AC incompletion, segmentation was carried out to distinguish the patients who received AC using the non-hierarchical k-means method. Three clusters were identified: cluster 1 (young patients with low AAC volume), cluster 2 (old patients with low AAC volume), and cluster 3 (old patients with high AAC volume). All statistical analyses were performed using JMP statistical software (JMP® 17; SAS Institute Inc., Cary, NC). Statistical significance was set at p < 0.05.

## Results

Patient characteristics 

Of the 257 patients who were diagnosed with pStage III CRC, 161 were included in the analysis, after excluding 81 who did not undergo AC, 10 who had recurrence during AC, and five who could not be followed up during AC. The baseline characteristics of the high and low AAC groups are shown in Table 1. Compared with the low AAC group, the high AAC group had a significantly higher proportion of patients with older age, male sex, higher ASA-PS, HT, and DM. No significant differences were found in malnutrition or systemic inflammation status, including mGPS, CONUT, GNRI, and NLR, between the two groups. In addition, there were no significant differences in tumor factors, including pStage and histopathological differentiation. Furthermore, there was no difference in the choice of regimen between the two groups. The rate of AC completion was lower in the high AAC group than in the low AAC group (72.9% vs. 90.3%; p < 0.01).

**Table 1:** Patient characteristics Categorical variables are expressed as numbers and percentages. Continuous variables are presented as medians with interquartile ranges. p-value < 0.05 was considered significant (chi-square test or nonparametric Mann-Whitney U test). AAC, abdominal aortic calcification; AC, adjuvant chemotherapy; ASA-PS, American Society of Anesthesiologists Physical Status Classification System; BMI, body mass index; CEA, carcinoembryonic antigen; CONUT, controlling nutritional status; DM, diabetes mellitus; GNRI, Geriatric Nutritional Risk Index; HL, hyperlipidemia; HT, hypertension; mGPS, modified Glasgow prognostic score; NLR, neutrophil-to-lymphocyte ratio; Por/muc, poorly/mucinous; pStage, pathological stage

	High AAC group (n = 48)	Low AAC group (n = 113)	p-value
Age (years)	71 (65-74)	61 (53-69)	<0.01
Gender (male/female)	38/10	52/61	<0.01
ASA-PS	-	-	<0.01
1	3 (6.3%)	33 (29.2%)	-
2	44 (91.7%)	79 (69.9%)	-
3	1 (2.1%)	1 (0.9%)	-
HT (+)	25 (52.1%)	26 (23.0%)	< 0.01
DM (+)	19 (39.6%)	28 (24.8%)	0.059
HL (+)	24 (50.0%)	54 (47.8%)	0.797
CONUT	-	-	0.799
Normal (0-1)	31 (64.6%)	71 (62.8%)	-
Mild (2-4)	17 (35.4%)	41 (36.3%)	-
Moderate (5-8)	0 (0.0%)	1 (0.9%)	-
High (9-)	0 (0.0%)	0 (0.0%)	-
GNRI	-	-	0.408
Normal (>98)	42 (87.5%)	93 (82.3%)	-
Mild (92-98)	2 (4.2%)	12 (10.6%)	-
Moderate (<92)	4 (8.3%)	8 (7.1%)	-
mGPS	-	-	0.696
0	43 (89.6%)	99 (87.6%)	-
1	4 (8.3%)	13 (11.5%)	-
2	1 (2.1%)	1 (0.9%)	-
NLR	2.51 (1.86-3.47)	2.23 (1.64-3.03)	0.145
Tumor location (colon/rectum)	28/20	58/55	0.415
pStage	-	-	0.582
Ⅲa	13 (27.1%)	38 (33.6%)	-
Ⅲb	31 (64.6%)	63 (55.8%)	-
Ⅲc	4 (8.3%)	12 (10.6%)	-
Por/muc (+)	3 (6.3%)	12 (10.6%)	0.383
CEA (ng/mL)	3.9 (2.4-9.1)	2.4 (1.7-6.0)	0.047
Postoperative complication (+)	12 (25.0%)	15 (13.3%)	0.069
Regimen	-	-	0.820
Fluoropyrimidine monotherapy	34 (70.8%)	78 (69.0%)	-
Oxaliplatin combination therapy	14 (29.2%)	35 (31.0%)	-
AC completion (+)	35 (72.9%)	102 (90.3%)	<0.01

Identification of risk factors for AC incompletion 

Univariate analysis revealed that older age (≥70 years) and high AAC (≥992 mm^3^) were risk factors for AC incompletion. The independent risk factors identified in the multivariate analysis were high AAC (OR, 3.00; 95% CI, 1.05-8.53; p = 0.040) and older age (OR, 4.85; 95% CI, 1.68-14.0; p < 0.01) (Table 2).

**Table 2:** Risk factors for adjuvant chemotherapy incompletion p-value < 0.05 was considered significant. AAC, abdominal aortic calcification; AC, adjuvant chemotherapy; ASA-PS, American Society of Anesthesiologists Physical Status Classification System; CONUT, controlling nutritional status; GNRI, Geriatric Nutritional Risk Index; mGPS, modified Glasgow prognostic score; NLR, neutrophil-to-lymphocyte ratio; OR, odds ratio; pStage, pathological stage

Factors	Univariate			Multivariate		
	OR	95% CI	p-value	OR	95% CI	p-value
Age (≥70 years)	4.68	1.88-11.6	<0.01	4.85	1.68-14.0	<0.01
Gender (male)	1.12	0.47-2.71	0.795	-	-	-
AAC (≥992 mm^3^)	3.44	1.41-8.39	<0.01	3.00	1.05-8.53	0.040
ASA-PS (≥2)	0.84	0.31-2.31	0.737	0.43	0.13-1.42	0.168
CONUT (≥5)	1.44	0.71-2.94	0.312	-	-	-
mGPS (≥1)	1.28	0.53-3.10	0.581	2.71	0.78-9.45	0.119
GNRI (<92)	0.81	0.09-6.87	0.845	-	-	-
NLR (≥4)	1.45	0.49-4.32	0.501	-	-	-
Tumor location (colon)	1.28	0.53-3.09	0.580	-	-	-
Histological type (Por/muc)	1.49	0.39-5.72	0.563	3.24	0.70-15.1	0.133
pStage (IIIb/IIIc)	1.47	0.55-3.95	0.448	-	-	-
Postoperative complication (+)	1.84	0.65-5.18	0.247	-	-	-
Fluoropyrimidine monotherapy	0.69	0.28-1.70	0.417	-	-	-

Risk ratios for AC incompletion according to the cluster analysis based on the age and AAC volume 

**Figure 1:**
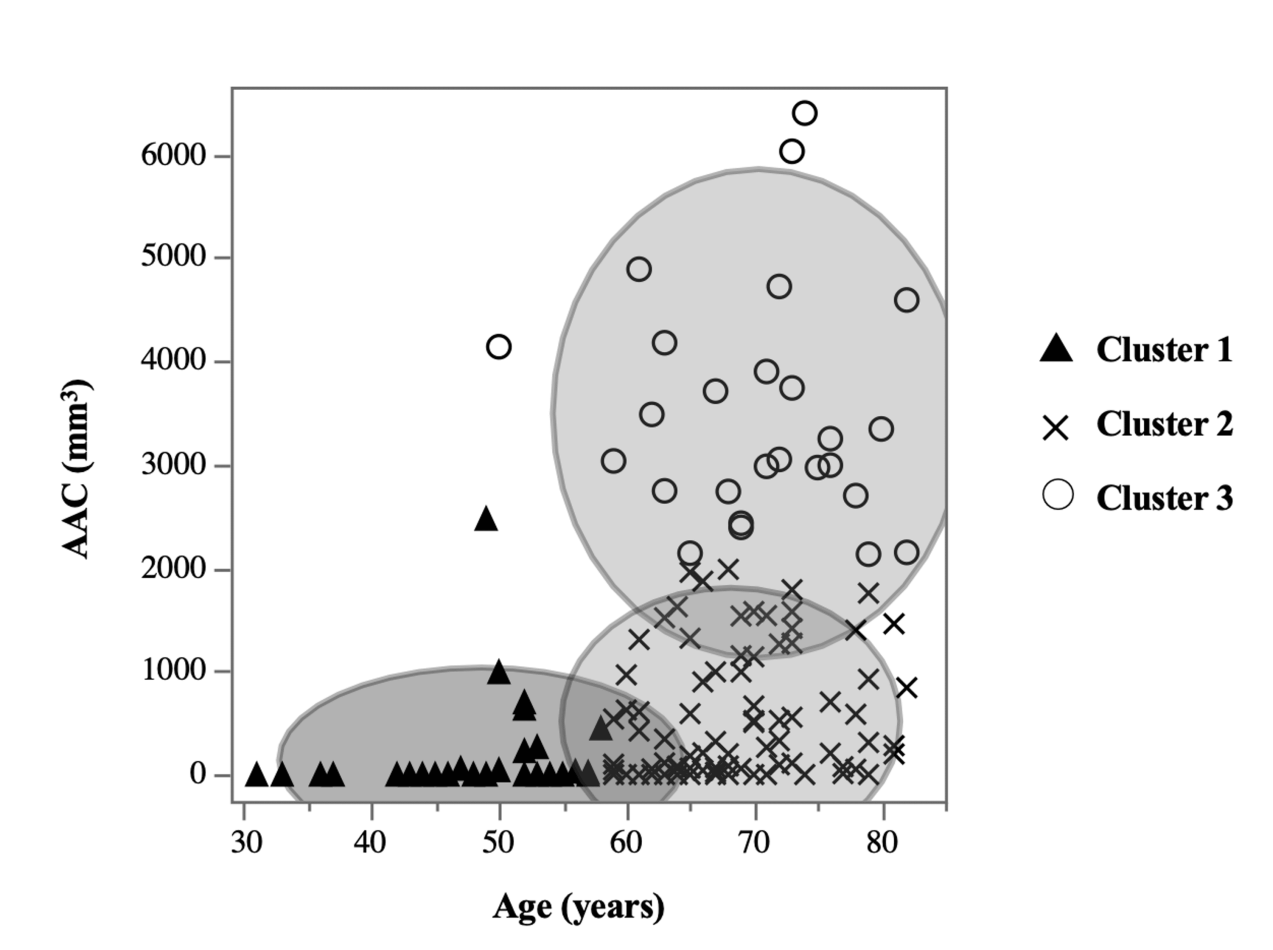
Cluster analysis based on the age and AAC volume A k-means cluster analysis is conducted to elucidate the effects of age and AAC volume on AC incompletion. This analysis uses both age and AAC volume to stratify into three distinct clusters. AAC, abdominal aortic calcification; AC, adjuvant chemotherapy

Subsequently, to elucidate the effects of age and AAC volume on AC incompletion, a k-means cluster analysis was performed. This analysis utilized both age and AAC volume for stratification into three distinct clusters (Figure 1): cluster 1 consisting of young age (50 (45-55) years) and low AAC (0 (0-30) mm^3^) (n = 43), cluster 2 consisting of old age (68 (63-72) years) and low AAC (208 (14-954) mm^3^) (n = 92), and cluster 3 consisting of old age (72 (65-76) years) and high AAC (3,147 (2,727-4,146) mm^3^) (n = 26). The odds ratio for AC incompletion increased with age and AAC volume (Table 3).

**Table 3:** Risk ratios for incompletion of adjuvant chemotherapy according to the cluster analysis based on the age and AAC volume Categorical variables are expressed as numbers and percentages. Continuous variables are presented as medians with interquartile ranges. p-value < 0.05 was considered significant. AAC, abdominal aortic calcification; CI, confidence interval; OR, odds ratio

	n = 161	Age (years)	AAC volume (mm^3^)	OR	95% CI	p-value
Cluster 1	43 (26.7%)	50 (45-55)	0 (0-30)	1 (reference)		
Cluster 2	92 (57.1%)	68 (63-72)	208 (14-954)	4.32	0.95-19.7	0.059
Cluster 3	26 (16.1%)	72 (65-76)	3,147 (2,727-4,146)	6.15	1.14-33.2	0.035

The severity and details of AEs between the high and low AAC groups

Table 4 shows the association between the severity or details of the AEs and AAC. There were no differences in the severity or details of the AEs between the high and low AAC groups. Nevertheless, the high AAC group significantly failed to complete AC because of AEs. These results suggest that patients with high AAC are more prone to treatment intolerance of AC, independent of AEs.

**Table 4:** Severity and details of adverse events between the high and low AAC groups Categorical variables are expressed as numbers and percentages. p-value < 0.05 was considered significant (chi-square test). AAC, abdominal aortic calcification; AC, adjuvant chemotherapy

	High AAC group (n = 48)	Low AAC group (n = 113)	p-value
The severity of adverse event	-	-	0.481
Grade 0	9 (18.8%)	12 (10.6%)	-
Grade 1	8 (16.7%)	23 (20.4%)	-
Grade 2	19 (39.6%)	53 (46.9%)	-
Grade 3	12 (25.0%)	25 (22.1%)	-
Grades 4-5	0 (0.0%)	0 (0.0%)	-
Hematological toxicity (≥grade 2)	12 (25.0%)	30 (26.6%)	0.838
Symptoms of digestive system (≥grade 2)	10 (20.8%)	21 (18.6%)	0.741
Hand-foot syndrome (≥grade 2)	12 (25.0%)	32 (28.3%)	0.666
Others (≥grade 2)	9 (18.8%)	19 (16.8%)	0.767

Perioperative changes in nutrition status, inflammatory markers, and renal function between the high and low AAC groups

**Figure 2:**
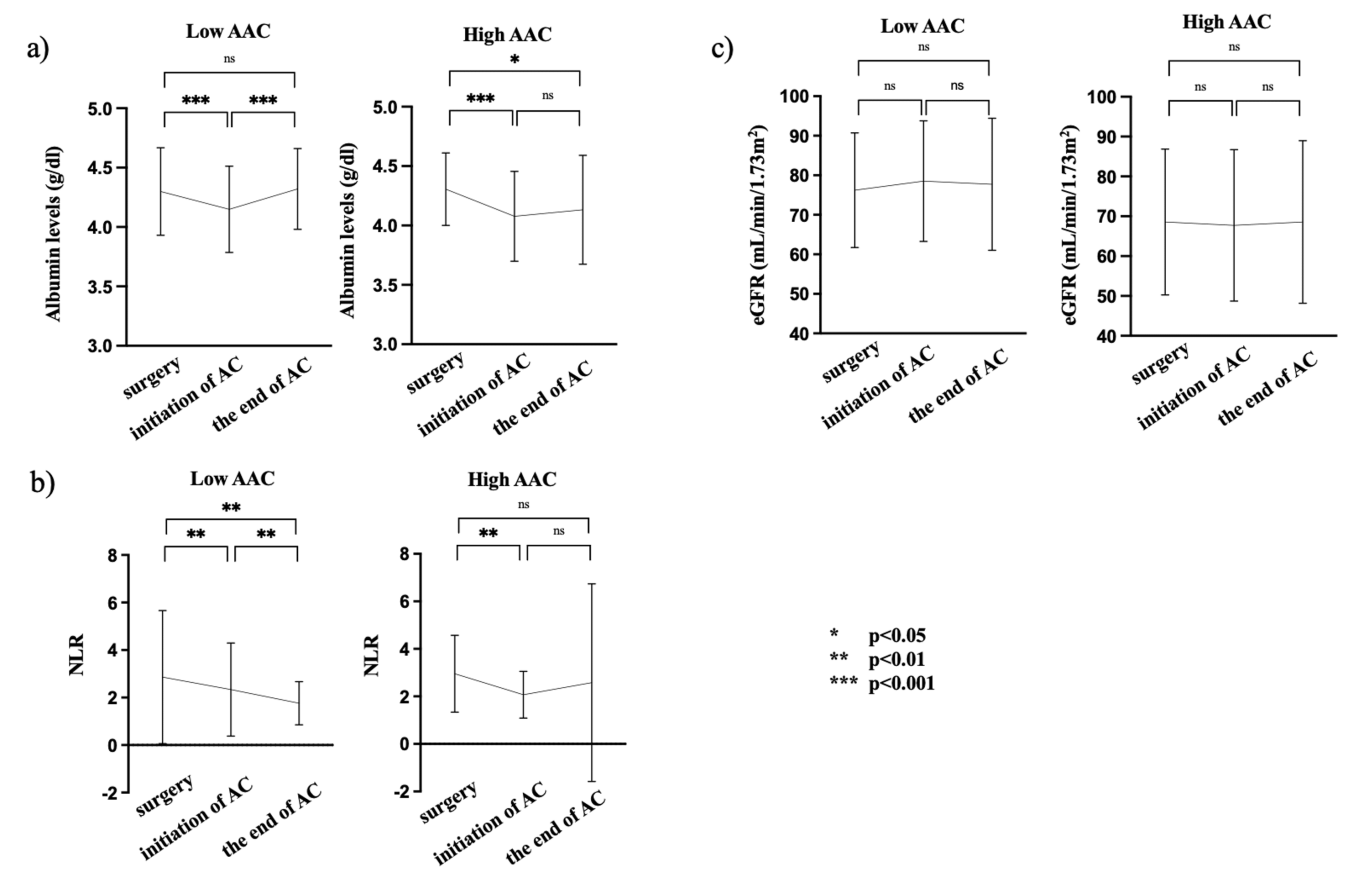
Perioperative changes in nutrition status, inflammatory marker, and renal function between the high and low AAC groups; a) albumin level, b) neutrophil-to-lymphocyte ratio, c) estimated glomerular filtration rate (eGFR) In the high AAC group, the decrease in nutritional status at the initiation of AC did not recover to the level at the time of surgery by the end of AC. Additionally, in the high AAC group, inflammatory markers at the end of AC did not show improvement compared to those at surgery. The eGFR exhibited no significant changes at any of the assessed time points in either group. Data analysis included paired t-tests (*p < 0.05, **p < 0.01, ***p < 0.001). AAC, abdominal aortic calcification; AC, adjuvant chemotherapy; eGFR, estimated glomerular filtration rate; NLR, neutrophil-to-lymphocyte ratio

We assessed the correlation between AAC and alterations in nutritional status, inflammatory markers, and renal function at three key time points: at the time of surgery, at the initiation of AC, and at the completion or discontinuation of AC. Upon initiation of AC, albumin levels declined compared to preoperative levels in both AAC groups. In the low AAC group, albumin levels at the end of AC showed improvement relative to the levels at initiation, eventually returning to preoperative levels (Figure 2a). Conversely, in the high AAC group, albumin levels at the end of AC remained stagnant compared to the levels at initiation and failed to recover to preoperative levels (Figure 2a). Similarly, NLR levels improved at the initiation of AC compared with preoperative levels in both AAC groups. Subsequent to AC, NLR levels decreased in the low AAC group compared with the initiation levels (Figure 2b). However, in the high AAC group, the NLR levels remained unchanged compared to the initiation level. In contrast, the estimated glomerular filtration rate (eGFR) exhibited no significant changes at any assessed time point, including at surgery, initiation of AC, and completion or discontinuation of AC in both AAC groups (Figure 2c). These findings suggest that high AAC was associated with diminished improvement in nutritional status or inflammatory markers following the introduction of AC.

## Discussion

This retrospective study aimed to identify potential predictors of AC incompletion in patients with stage III CRC, with a specific focus on the role of AAC as an indicator of AC incompletion. A notable finding was that high AAC volume was a significant predictor of AC incompletion in patients with stage III CRC. This finding underscores the comprehensive reflection of systemic conditions such as advanced age and comorbidities by AAC. Importantly, AAC was robustly associated with the critical outcomes of chemotherapy discontinuation in patients with CRC. This association suggests that AAC could effectively bridge the clinical gap by identifying risk factors that may impede AC completion prior to its initiation. Additionally, there is an increased risk of AC incompletion in patients with stage III CRC as both age and AAC volume increase. To the best of our knowledge, this is the first study to establish a direct link between AAC and AC incompletion in patients with stage III CRC who commenced AC. These findings present AAC as a straightforward and immediately applicable metric in clinical settings.

The identification of clinically effective biomarkers for exploring the sustainability of AC in the older population presents a significant challenge to clinical practice. One study reported that there was no age requirement for postoperative AC, and aggregated analyses of randomized-controlled trials conducted in both the United States and Europe have demonstrated comparable effectiveness in terms of recurrence prevention and survival extension between patients aged ≥70 years and those aged <60 years [20]. Our previous study also indicated that completion of AC may contribute to improved long-term prognosis, even in patients aged ≥ 80 years with stage III CRC [21]. However, older age is considered an important factor in the introduction and continuation of AC by many clinicians and patients [22,23]. Increased age may also be associated with increased frailty and decreased tolerance to chemotherapy [24]. Although the decision on AC introduction or discontinuation should be made carefully, considering not only age but also major organ functions, general health, and condition, no clinically useful biomarkers have yet been identified. Recently, geriatric assessment (GA) has been shown to be a useful method for measuring physical function, cognitive function, nutritional status, social factors, and family environment and has been reported to predict the completion of chemotherapy [25,26]. Although GA has proven useful for characterizing health and functional impairments potentially associated with oncological outcomes [27], it requires considerable time and human resources [28]. Therefore, it is necessary to establish a simpler biomarker for predicting AC completion than that for predicting GA. AAC is a well-known risk marker of cardiovascular diseases and is associated with hyperphosphatemia, diabetes, chronic inflammation, and chronic kidney disease [29]. A recent study indicated that AAC severity independently correlated with an increased risk of pre-frailty or frailty in a dose-responsive relationship [9]. Furthermore, AAC volume can be calculated and quantified accurately, automatically, and rapidly using preoperative CT-based examination [30]. Our findings indicate that AAC volume is a specific predictor of AC completion as well as chronological age. This distinction is critical, as even among older patients, there is considerable variation in the overall health status and vulnerability, which are influenced by factors such as the extent of comorbidities and tumor progression. However, these variations cannot be accurately gauged using age alone. Consequently, the measurement of AAC volume is anticipated to offer a more objective reflection of the systemic condition and tolerance to AC in patients.

Contrary to expectations, high AAC was not associated with the severity of AEs but the risk of AC incompletion in stage III CRC patients in this study. High AAC can be related to the patients’ adverse condition that cannot be assessed by the Common Terminology Criteria for Adverse Events (CTCAE) after AC introduction. Therefore, we focused on the association of AAC with alterations in nutritional status and inflammatory markers following the introduction of AC. Our findings suggest that higher levels of AAC are linked to a reduced improvement in nutritional status or inflammatory markers after the administration of AC, which can potentially contribute to AC incompletion independent of the AE severity. Thus, AAC could serve as an indicative marker of patient-specific susceptibility to the tolerance of AC.

This study had some limitations that should be considered when interpreting our findings. Specifically, the retrospective and non-randomized study design must be mentioned. The small sample size of patients at a single center may also weaken the conclusion. In this study, AAC was quantified using the AZE Virtual Place Lexus64 Anatomia software. However, as this software is not widely accessible, the generalizability and reproducibility of the study's findings may be limited. This constraint should be considered when interpreting the results. Future prospective studies involving a larger number of patients with high AAC are required to analyze the effects of AC on clinical outcomes.

## Conclusions

This study demonstrated that high AAC was a potential risk marker for predicting AC incompletion in patients with stage III CRC before introducing AC. Patients with high AAC are more prone to treatment intolerance of AC irrespective of the occurrence of adverse events. This intolerance may be associated with diminished improvement in nutritional status or inflammatory markers following the introduction of AC. Given that elevated AAC correlates with advanced age and frailty, high AAC volume may help us to follow up more closely with patients who have a potential risk of AC incompletion before AC introduction. Based on the results of this study, adjusting the chemotherapy dose intensity and scheduling, while ensuring treatment effectiveness, could be an important strategy for patients with high AAC volume to minimize treatment interruptions. This personalized intervention may help improve the completion rates of AC and clinical outcomes in patients with high AAC volume.

## References

[1] Morgan E, Arnold M, Gini A (2023). Global burden of colorectal cancer in 2020 and 2040: incidence and mortality estimates from GLOBOCAN. Gut.

[2] Knapen DG, Cherny NI, Zygoura P (2020). Lessons learnt from scoring adjuvant colon cancer trials and meta-analyses using the ESMO-Magnitude of Clinical Benefit Scale V.1.1. ESMO Open.

[3] Yoshino T, Arnold D, Taniguchi H (20181). Pan-Asian adapted ESMO consensus guidelines for the management of patients with metastatic colorectal cancer: a JSMO-ESMO initiative endorsed by CSCO, KACO, MOS. SSO and TOS. Ann Oncol.

[4] Shahrokni A, Kim SJ, Bosl GJ, Korc-Grodzicki B (2017). How we care for an older patient with cancer. J Oncol Pract.

[5] Abdel-Rahman O, Tang PA, Koski S (2021). Hospitalizations among early-stage colon cancer patients receiving adjuvant chemotherapy: a real-world study. Int J Colorectal Dis.

[6] Criqui MH, Denenberg JO, McClelland RL (2014). Abdominal aortic calcium, coronary artery calcium, and cardiovascular morbidity and mortality in the Multi-Ethnic Study of Atherosclerosis. Arterioscler Thromb Vasc Biol.

[7] Zhang K, Gao J, Chen J, Liu X, Cai Q, Liu P, Huang H (2016). MICS, an easily ignored contributor to arterial calcification in CKD patients. Am J Physiol Renal Physiol.

[8] Stenvinkel P, Heimbürger O, Lindholm B, Kaysen GA, Bergström J (2000). Are there two types of malnutrition in chronic renal failure? Evidence for relationships between malnutrition, inflammation and atherosclerosis (MIA syndrome). Nephrol Dial Transplant.

[9] Lee SY, Chao CT, Huang JW, Huang KC (2020). Vascular calcification as an underrecognized risk factor for frailty in 1783 community-dwelling elderly individuals. J Am Heart Assoc.

[10] Smith C, Sim M, Dalla Via J (2024). Extent of abdominal aortic calcification is associated with incident rapid weight loss over 5 years: the Perth Longitudinal Study of Ageing Women. Arterioscler Thromb Vasc Biol.

[11] Ide R, Ohira M, Imaoka Y (2023). Impact of abdominal aortic calcification on chronic kidney disease after liver transplantation: a retrospective study. Transplant Proc.

[12] Dees EC, O'Reilly S, Goodman SN (2000). A prospective pharmacologic evaluation of age-related toxicity of adjuvant chemotherapy in women with breast cancer. Cancer Invest.

[13] Hackett NJ, De Oliveira GS, Jain UK, Kim JY (2015). ASA class is a reliable independent predictor of medical complications and mortality following surgery. Int J Surg.

[14] Hashiguchi Y, Muro K, Saito Y (2020). Japanese Society for Cancer of the Colon and Rectum (JSCCR) guidelines 2019 for the treatment of colorectal cancer. Int J Clin Oncol.

[15] Agatston AS, Janowitz WR, Hildner FJ (1990). Quantification of coronary artery calcium using ultrafast computed tomography. J Am Coll Cardiol.

[16] Hirashima K, Watanabe M, Shigaki H (2014). Prognostic significance of the modified Glasgow prognostic score in elderly patients with gastric cancer. J Gastroenterol.

[17] Li L, Wang H, Yang J (2018). Geriatric nutritional risk index predicts prognosis after hepatectomy in elderly patients with hepatitis B virus-related hepatocellular carcinoma. Sci Rep.

[18] Elghiaty A, Kim J, Jang WS (2019). Preoperative controlling nutritional status (CONUT) score as a novel immune-nutritional predictor of survival in non-metastatic clear cell renal cell carcinoma of ≤ 7 cm on preoperative imaging. J Cancer Res Clin Oncol.

[19] Guthrie GJ, Charles KA, Roxburgh CS, Horgan PG, McMillan DC, Clarke SJ (2013). The systemic inflammation-based neutrophil-lymphocyte ratio: experience in patients with cancer. Crit Rev Oncol Hematol.

[20] Sargent DJ, Goldberg RM, Jacobson SD (2001). A pooled analysis of adjuvant chemotherapy for resected colon cancer in elderly patients. N Engl J Med.

[21] Mochizuki T, Shimomura M, Nakahara M (2024). Survival outcomes of patients with stage III colorectal cancer aged ≥ 80 years who underwent curative resection: the HiSCO-04 prospective cohort study. Int J Clin Oncol.

[22] Kim HH, Ihn MH, Lee YH, Lee J, Yun S, Cho SW (2020). Effect of age on laparoscopic surgery and postoperative chemotherapy in elderly patients with colorectal cancer. Ann Coloproctol.

[23] Badic B, Oguer M, Cariou M (2021). Prognostic factors for stage III colon cancer in patients 80 years of age and older. Int J Colorectal Dis.

[24] Dobie SA, Baldwin LM, Dominitz JA, Matthews B, Billingsley K, Barlow W (2006). Completion of therapy by Medicare patients with stage III colon cancer. J Natl Cancer Inst.

[25] Aaldriks AA, Maartense E, Nortier HJ (2016). Prognostic factors for the feasibility of chemotherapy and the Geriatric Prognostic Index (GPI) as risk profile for mortality before chemotherapy in the elderly. Acta Oncol.

[26] von Gruenigen VE, Huang HQ, Beumer JH (2017). Chemotherapy completion in elderly women with ovarian, primary peritoneal or fallopian tube cancer - an NRG oncology/Gynecologic Oncology Group study. Gynecol Oncol.

[27] Wildiers H, Heeren P, Puts M (2014). International Society of Geriatric Oncology consensus on geriatric assessment in older patients with cancer. J Clin Oncol.

[28] Caillet P, Laurent M, Bastuji-Garin S (2014). Optimal management of elderly cancer patients: usefulness of the Comprehensive Geriatric Assessment. Clin Interv Aging.

[29] Kiu Weber CI, Duchateau-Nguyen G, Solier C, Schell-Steven A, Hermosilla R, Nogoceke E, Block G (2014). Cardiovascular risk markers associated with arterial calcification in patients with chronic kidney disease Stages 3 and 4. Clin Kidney J.

[30] Yoon YE, Han WK, Lee HH (2016). Abdominal aortic calcification in living kidney donors. Transplant Proc.

